# Identifying frailty trajectories in older patients with acute myocardial infarction using structural entropy clustering: a prospective cohort study

**DOI:** 10.1186/s12877-026-07614-4

**Published:** 2026-05-14

**Authors:** Tongtong Zhang, Haoran Yang, Xiaoping Lou, Chao Lan, Naifu Tang, Bo Li

**Affiliations:** 1 Department of Emergency Medicine, The First Affiliated Hospital of Zhengzhou University, No.1 Longhu Zhonghuan Road, Zhengzhou, Henan 450052 China; 2 Big Data Institute, Central South University, No.932 South Lushan Road, Changsha, Hunan 410083 China; 3 Nursing Department, The First Affiliated Hospital of Zhengzhou University, No.1 Longhu Zhonghuan Road, Zhengzhou, Henan 410083 China

**Keywords:** Acute Myocardial Infarction, Cluster Analysis, Geriatric Assessment, Frailty, Nursing Care

## Abstract

**Background:**

Frailty significantly complicates clinical outcomes in older adults with acute myocardial infarction, yet its progression is dynamic and heterogeneous. This study aimed to identify distinct frailty trajectory patterns and their predictors using a machine learning approach, to support evidence-based nursing interventions.

**Methods:**

A prospective cohort study was conducted, enrolling 583 older adults with acute myocardial infarction hospitalized between March 2023 and March 2024. We collected multidimensional clinical, physiological, psychological, and functional data at six time points over a one-year follow-up period. A patient similarity network was constructed from these longitudinal data, and the Structural Entropy Clustering algorithm was employed to identify frailty trajectory groups. Group differences were analyzed using ANOVA and Tukey’s post hoc tests, while multinomial logistic regression was used to determine key predictors of trajectory membership.

**Results:**

Four distinct frailty trajectories were identified: “Rapidly Worsening Frailty” ($n=78$, 13.4%), “Stable Non-Frail” ($n=261$, 44.7%), “Slowly Progressive Frailty” ($n=218$, 37.4%), and “Improving Frailty” ($n=26$, 4.5%). Significant differences were observed among the groups in functional status, psychological scores, nutritional status, left ventricular ejection fraction, and Charlson Comorbidity Index ($p<0.05$). Multivariate analysis revealed that lower functional status (Modified Barthel Index per 10-point decrease: $OR=9.34$, 95% CI: 7.37–11.82, $p<0.05$) and advanced age ($OR=1.07$, $p<0.05$) were strong predictors for the “Rapidly Worsening Frailty” trajectory, while psychological factors including anxiety ($OR=2.33$, $p<0.05$) and depression ($OR=2.50$, $p<0.05$) were significant predictors for the “Slowly Progressive Frailty” trajectory.

**Conclusions:**

Frailty progression following acute myocardial infarction is heterogeneous, and distinct trajectory patterns can be identified using structural entropy clustering. These findings may support the development of differentiated nursing strategies for early identification of high-risk individuals, pending validation in multicenter settings.

## Background

Acute myocardial infarction (AMI) is a leading cause of morbidity and mortality in older adults, with those aged 65 and over accounting for over 60% of AMI cases in China [1]. The clinical management of these patients is further complicated by frailty, an age-related syndrome characterized by decreased physiological reserve and increased vulnerability to stressors [2, 3]. Frailty elevates the risk of hospitalization, falls, and mortality [4–6], while negatively impacting quality of life [7]. Frailty progression after AMI has also been identified as an independent risk factor for adverse outcomes [8, 9], underscoring the need for targeted nursing strategies in this population [10, 11].

Frailty post-AMI is not a uniform process of decline but a dynamic condition in which patients follow distinct trajectories associated with different prognoses [12–14]. Such longitudinal monitoring supports individualized approaches to frailty management [15, 16]. However, a gap persists in the availability of practical tools to stratify patients into these trajectories, as traditional clinical assessments struggle to capture the multidimensional and dynamic nature of frailty [17, 18].

To address this, the present study applied the Structural Entropy Clustering (SEC) algorithm to identify heterogeneous frailty trajectories in older adults with AMI. Compared with conventional methods such as k-means [19], Latent Class Growth Modeling [20], and Hidden Markov Models [21], SEC is a non-parametric approach that constructs a patient similarity network and partitions it by minimizing structural entropy, without requiring assumptions about data distribution [22]. Using multidimensional clinical indicators, this study aimed to stratify patients into clinically relevant trajectory groups and identify key predictors of trajectory membership, with the goal of informing differentiated nursing interventions [23–25].

## Methods

### Study design and setting

We conducted a prospective, longitudinal cohort study at a single tertiary hospital in China. This study was conducted and reported in accordance with the Strengthening the Reporting of Observational Studies in Epidemiology (STROBE) statement.

### Ethical considerations

The study protocol was approved by the Research and Clinical Trial Ethics Committee of the First Affiliated Hospital of Zhengzhou University (Approval ID: 2023-KY-0204-002) and adhered to the ethical guidelines of the Declaration of Helsinki. Written informed consent was obtained from all participants prior to enrollment. All data used for analysis were fully de-identified to ensure patient confidentiality.

### Study population and data source

Patients diagnosed with AMI were recruited using a convenience sampling method between March 2023 and September 2023. An initial cohort of 615 patients was enrolled; after a one-year follow-up period, 32 patients were lost to follow-up (attrition rate of 5.2%). For the remaining 583 participants who completed the study (Fig. 4 in Appendix A), missing values for longitudinal variables were handled using Multiple Imputation with Chained Equations [26]. Eligible patients aged 65 years or older were prospectively screened by a trained research nurse within 48 hours of admission and enrolled prior to discharge. The baseline assessment (T1) was conducted on the second day post-admission, once clinical stability was achieved.

Inclusion criteria for the study were: a confirmed diagnosis of AMI based on clinical, electrocardiographic, and biochemical criteria; age 65 years or older; the availability of complete clinical and demographic data; and the ability to communicate effectively to participate in follow-up assessments. Exclusion criteria included: (1) Severe cognitive impairment that precluded effective communication, as evaluated and determined by a qualified neurologist through standardized clinical cognitive assessment; (2) Severe comorbidities that significantly impacted survival prognosis, such as metastatic cancer or end-stage renal disease requiring dialysis; (3) Participation in other interventional studies that could affect frailty status; (4) Recent (within the last 3 months) use of systemic steroids, as these medications may confound frailty assessment through effects on muscle mass and function.

### Measurements and data collection

Data were collected by trained investigators using standardized assessments. Baseline data (predictor variables) were collected during hospitalization. Longitudinal follow-up data were collected at six key time points: two days post-admission (T1), and at 1, 3, 6, 9, and 12 months post-discharge (T2-T6).

At each follow-up, frailty was assessed using the validated Fried Frailty Phenotype [27], which comprises five components: unintentional weight loss, exhaustion, weakness (grip strength), slowness (gait speed), and low physical activity. Patients were classified as non-frail (0 criteria), pre-frail (1-2 criteria), or frail ($\ge$3 criteria). Additional measures were also collected at each follow-up, including functional status assessed with the Modified Barthel Index (MBI) to measure activities of daily living [28]; comorbidity burden quantified using the Charlson Comorbidity Index (CCI) [29]; psychological status evaluated via the Zung Self-Rating Anxiety and Depression Scales; and key clinical parameters such as left ventricular ejection fraction (LVEF), long-term medication count, and sleep score.

### Trajectory identification using structural entropy clustering

To identify distinct frailty trajectories, we employed the SEC algorithm. This process involved two steps. First, patient characteristics (static factors and dynamic time-series data) were standardized, and pairwise distances were computed using Dynamic Time Warping (DTW) for longitudinal variables. The total distance was calculated as a weighted sum of static and dynamic components:

1 $$\begin{aligned} D_{\text {total}} = \alpha D_{\text {static}} + (1-\alpha ) D_{\text {DTW}} \end{aligned}$$

where $\alpha$ was determined via cross-validation. The distance matrix was then converted to a similarity matrix using a Gaussian kernel. Second, the SEC algorithm was applied to partition the resulting patient similarity network by minimizing structural entropy:

2 $$\begin{aligned} H(G) = -\sum \limits _{c=1}^{C} p_c \log p_c \end{aligned}$$

where $p_c$ is the probability distribution of nodes belonging to cluster *c*, and *C* is the total number of clusters. The final cluster solution was selected based on entropy minimization and clinical interpretability, and was compared against k-means as an alternative method.

### Statistical analysis

Following the identification of frailty trajectories, we compared clinical variables and outcomes across the clusters. Continuous variables were summarized as means ± standard deviations (SD), while categorical variables were presented as counts and percentages (%). We used analysis of variance (ANOVA) for continuous variables and the $\chi ^2$ test for categorical variables to evaluate intergroup differences. When ANOVA was significant, Tukey’s Honest Significant Difference (HSD) test was performed for post hoc pairwise comparisons. Multinomial logistic regression was used to identify significant predictors of trajectory membership.

All statistical analyses were conducted using Python (version 3.9) with its core libraries (NumPy, pandas, scikit-learn, scipy). A two-tailed $p < 0.05$ was considered statistically significant.

### Predictive model development and validation

To assess the performance of the multinomial logistic regression model in predicting trajectory membership, we employed a 5-fold stratified cross-validation procedure (Table 4 in Appendix A). The dataset was partitioned into 5 subsamples, ensuring that the proportion of each trajectory class was maintained across each fold. For each fold, one subsample was used as the test set, and the remaining four were used for training. We calculated the average accuracy, precision, recall, and F1-score across the 5 folds. Additionally, the Area Under the Receiver Operating Characteristic Curve was calculated for each class to evaluate the model’s discriminatory power.

## Results

### Cohort characteristics

Of the 615 older patients with AMI who were initially enrolled, 583 (94.8%) completed the one-year follow-up and were included in the final analysis. The mean age of the participants was 74.32 ± 10.21 years. There were no significant differences in baseline demographic or clinical variables between patients who completed follow-up and those who were lost to follow-up ($n=32$) ($p > 0.05$).

### Identification of frailty trajectories

**Fig. 1:**
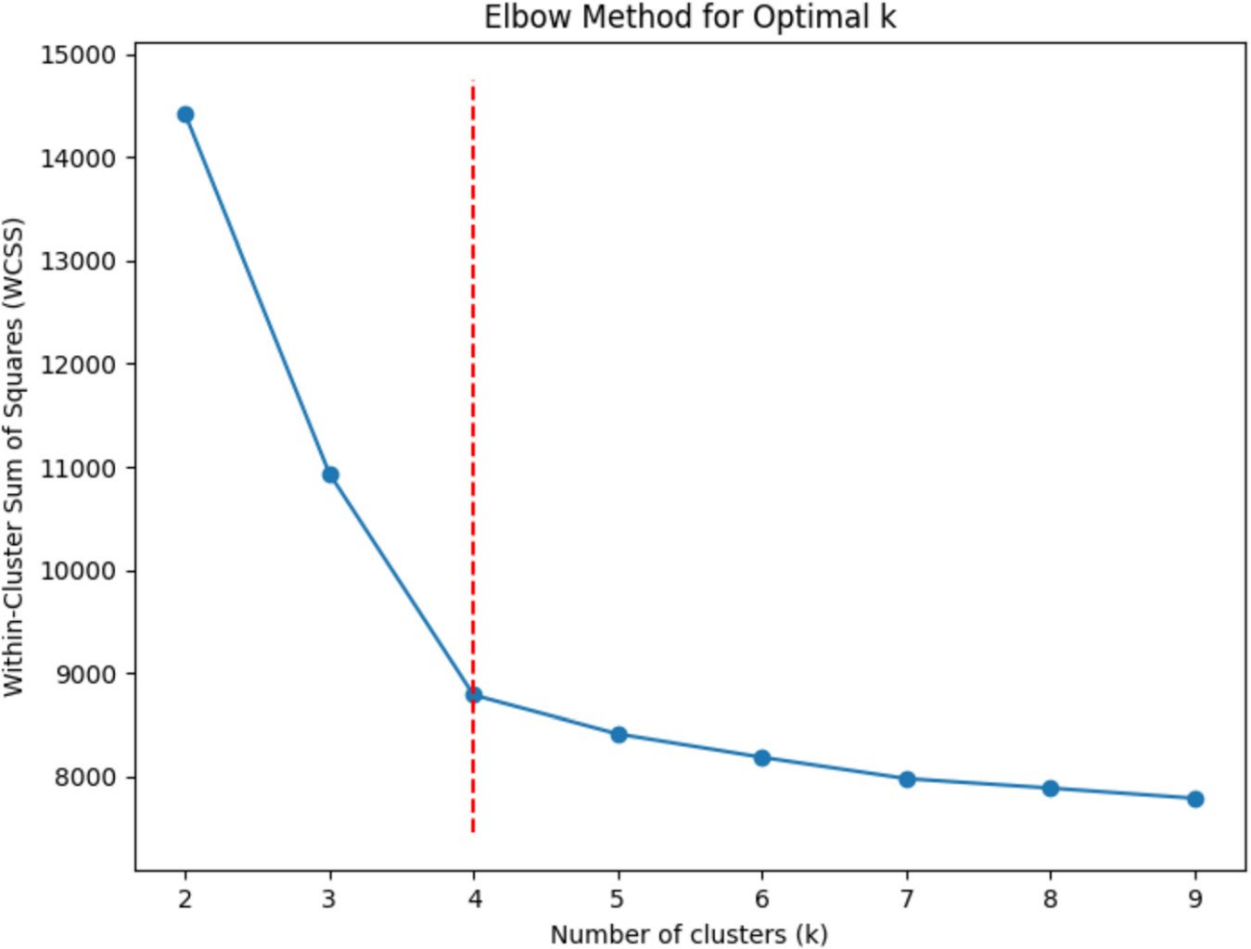
Elbow plot showing the leveling-off point at k=4, indicating the optimal number of clusters

To identify heterogeneous patterns of frailty progression, the SEC algorithm was applied to the longitudinal data of the 583 patients. The elbow plot of model fit indices suggested that a four-cluster solution was optimal, as performance gains plateaued beyond this point (Fig. 1).

**Fig. 2:**
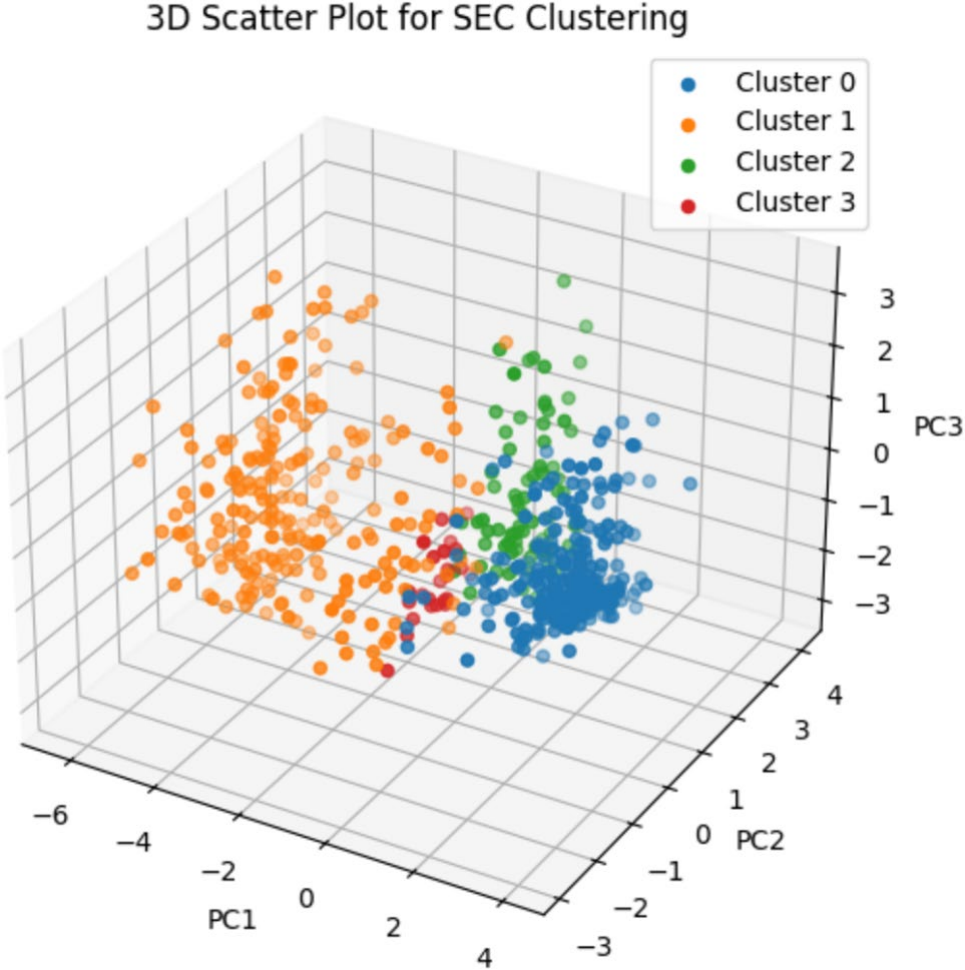
Three-dimensional scatter plot of the four frailty trajectories identified by the SEC algorithm

Based on this, the SEC algorithm successfully classified all patients into four clinically distinguishable trajectories, with clear separation shown in the three-dimensional scatter plot (Fig. 2). These trajectories were labeled as: Rapidly Worsening Frailty ($n=78$, 13.4%), Slowly Progressive Frailty ($n=218$, 37.4%), Improving Frailty ($n=26$, 4.5%), and Stable Non-Frail ($n=261$, 44.7%).

### Clinical characterization of trajectories

The four trajectories exhibited significant differences in both baseline characteristics and one-year outcomes (Table 1). At baseline, the Rapidly Worsening Frailty group had the highest comorbidity burden (CCI $3.03 \pm 0.97$) and Killip class ($2.44 \pm 0.59$), whereas the Stable Non-Frail group had the most favorable clinical profile.

**Table 1:** Mean comparison of baseline symptom scores and one-year outcomes across the four frailty trajectories

Features	Frailty Groups				ANOVA		Post hoc pairwise comparison^*a*^					
	Rapidly Worsening Frailty ($\boldsymbol{n = 78}$)	Slowly Progressive Frailty ($\boldsymbol{n = 218}$)	Improving Frailty ($\boldsymbol{n = 26}$)	Stable Non-Frail ($\boldsymbol{n = 261}$)	F statistic	*p*-value	1 vs 2	1 vs 3	1 vs 4	2 vs 3	2 vs 4	3 vs 4
Baseline scores												
Long-term Medication	$4.60 \pm 1.11$	$5.31 \pm 1.02$	$5.58 \pm 0.95$	$5.02 \pm 1.05$	11.11	$<0.05$	$<0.05$	$<0.05$	$<0.05$	0.183	0.003	0.008
Sleep Score	$9.05 \pm 2.30$	$8.10 \pm 2.03$	$7.81 \pm 1.79$	$6.89 \pm 2.11$	26.62	$<0.05$	$<0.05$	$<0.05$	$<0.05$	0.442	$<0.05$	$<0.05$
LVEF	$47.76 \pm 4.40$	$47.14 \pm 4.73$	$46.88 \pm 3.81$	$54.41 \pm 4.96$	108.76	$<0.05$	0.298	0.336	$<0.05$	0.757	$<0.05$	$<0.05$
Killip	$2.44 \pm 0.59$	$2.07 \pm 0.58$	$1.69 \pm 0.47$	$1.57 \pm 0.56$	60.42	$<0.05$	$<0.05$	$<0.05$	$<0.05$	$<0.05$	$<0.05$	0.213
CCI	$3.03 \pm 0.97$	$2.26 \pm 0.75$	$1.38 \pm 0.70$	$0.67 \pm 0.72$	271.29	$<0.05$	$<0.05$	$<0.05$	$<0.05$	$<0.05$	$<0.05$	$<0.05$
One-year outcomes												
Long-term Medication	$7.67 \pm 1.55$	$6.62 \pm 1.39$	$6.01 \pm 0.90$	$5.16 \pm 1.13$	152.82	$<0.05$	$<0.05$	$<0.05$	$<0.05$	$<0.05$	$<0.05$	$<0.05$
Sleep Score	$7.96 \pm 2.05$	$8.23 \pm 1.97$	$6.06 \pm 2.09$	$5.21 \pm 1.22$	110.72	$<0.05$	$<0.05$	$<0.05$	$<0.05$	0.519	$<0.05$	$<0.05$
LVEF	$41.14 \pm 7.15$	$45.35 \pm 5.43$	$55.78 \pm 4.46$	$57.43 \pm 5.73$	304.12	$<0.05$	$<0.05$	$<0.05$	$<0.05$	$<0.05$	$<0.05$	$<0.05$
Killip	$2.25 \pm 0.60$	$1.96 \pm 0.66$	$1.12 \pm 0.32$	$1.12 \pm 0.34$	263.69	$<0.05$	$<0.05$	$<0.05$	0.936	$<0.05$	$<0.05$	$<0.05$
CCI	$3.28 \pm 0.96$	$2.99 \pm 0.90$	$2.54 \pm 1.07$	$0.90 \pm 0.77$	320.12	$<0.05$	0.017	0.062	$<0.05$	$<0.05$	$<0.05$	$<0.05$

^*a*^Post hoc pairwise comparison using Tukey HSD

At one year, these differences intensified. The Rapidly Worsening group showed the lowest LVEF ($41.14 \pm 7.15$) and highest medication load, confirming continued deterioration. Conversely, the Improving Frailty group demonstrated recovery in cardiac function (LVEF increasing from $46.88 \pm 3.81$ to $55.78 \pm 4.46$), reaching levels comparable to the Stable Non-Frail group ($57.43 \pm 5.73$). The Slowly Progressive group occupied an intermediate position across all measures.

The Improving Frailty group ($n=26$, 4.5%) showed a distinct recovery pattern despite an initial clinical profile similar to the other frailty groups (baseline LVEF $46.88 \pm 3.81$; Killip class $1.69 \pm 0.47$, comparable to the Slowly Progressive group), with marked improvement across outcomes at one year. Cardiac function improved markedly, with mean LVEF increasing by 8.9 percentage points to $55.78 \pm 4.46$, a level statistically indistinguishable from the Stable Non-Frail group ($57.43 \pm 5.73$). Simultaneously, Killip class normalized to $1.12 \pm 0.32$, matching the most favorable outcome achieved by the Stable Non-Frail group, and sleep quality improved from $7.81 \pm 1.79$ to $6.06 \pm 2.09$. This trajectory provides evidence that frailty after AMI is not invariably progressive, and that a meaningful subset of patients retains substantial recovery potential when provided with appropriate clinical support.

**Fig. 3:**
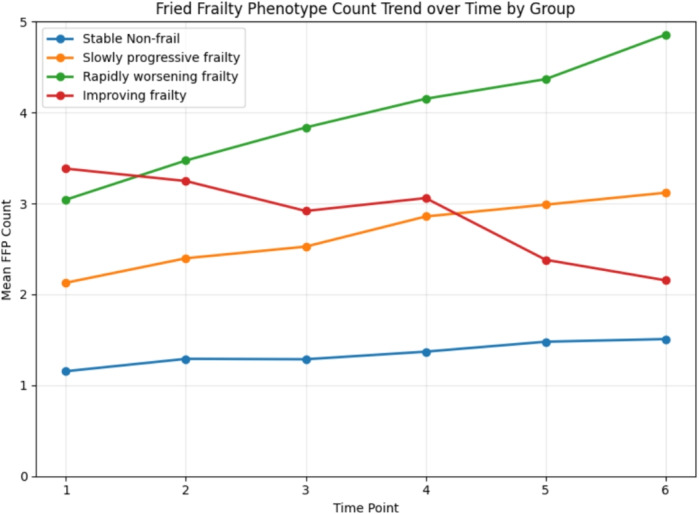
Trends in Fried Frailty Phenotype Count across the four frailty trajectories over one year

**Fig. 4:**
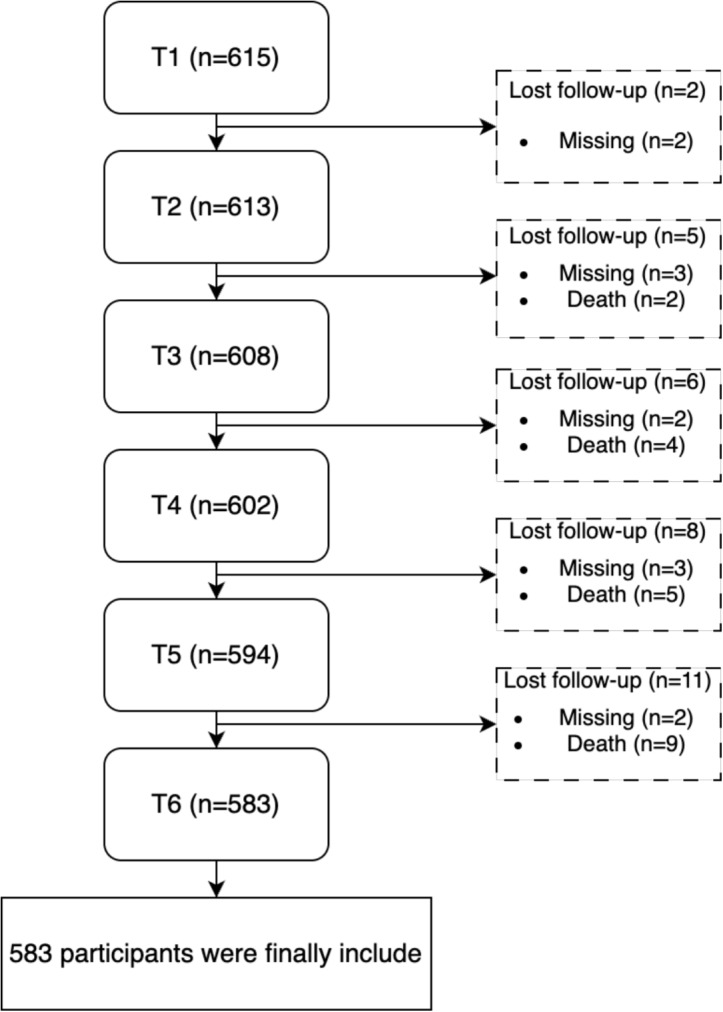
Flowchart outlining patient enrollment and lost to follow-up

Longitudinal trends in Fried Frailty Phenotype Count showed distinct patterns for each trajectory (Fig. 3).

### Predictors of trajectory membership

We performed a multinomial logistic regression analysis to identify baseline predictors associated with trajectory membership (Table 2).

**Table 2:** Multinomial Logistic Regression Analysis of Frailty Trajectory Predictors

Variables	$\boldsymbol{\beta }$ (SE)	OR (95% CI)	*p*-value
Rapidly worsening frailty^*a*^			
Age, years	0.071 (0.016)	1.07 (1.04-1.11)	<0.05
Male sex	-0.401 (0.160)	0.67 (0.49-0.92)	<0.05
Urban residence	-1.066 (0.162)	0.34 (0.25-0.47)	<0.05
Drinking history	-0.182 (0.195)	0.83 (0.57-1.22)	0.350
Smoking history	-0.174 (0.230)	0.84 (0.54-1.32)	0.450
Poor nutritional status	-0.218 (0.240)	0.80 (0.50-1.29)	0.364
Modified Barthel Index^*b*^	2.234 (0.120)	9.34 (7.37-11.82)	<0.05
Anxiety	0.246 (0.217)	1.28 (0.84-1.96)	0.258
Depression	-0.210 (0.260)	0.81 (0.49-1.35)	0.420
Slowly progressive frailty^*a*^			
Age, years	0.052 (0.015)	1.05 (1.02-1.08)	<0.05
Male sex	-1.113 (0.167)	0.33 (0.24-0.46)	<0.05
Urban residence	-0.921 (0.170)	0.40 (0.29-0.56)	<0.05
Drinking history	0.386 (0.187)	1.47 (1.02-2.12)	<0.05
Smoking history	0.474 (0.196)	1.61 (1.09-2.36)	<0.05
Poor nutritional status	1.283 (0.181)	3.61 (2.53-5.14)	<0.05
Modified Barthel Index^*b*^	2.108 (0.112)	8.23 (6.60-10.26)	<0.05
Anxiety	0.844 (0.194)	2.33 (1.59-3.40)	<0.05
Depression	0.914 (0.205)	2.50 (1.67-3.73)	<0.05
Improving frailty^*a*^			
Age, years	0.066 (0.029)	1.07 (1.01-1.13)	<0.05
Male sex	-0.812 (0.302)	0.44 (0.25-0.80)	<0.05
Urban residence	-0.944 (0.322)	0.39 (0.21-0.73)	<0.05
Drinking history	1.222 (0.304)	3.39 (1.87-6.15)	<0.05
Smoking history	1.354 (0.301)	3.87 (2.15-6.99)	<0.05
Poor nutritional status	1.444 (0.306)	4.24 (2.33-7.72)	<0.05
Modified Barthel Index^*b*^	1.578 (0.162)	4.85 (3.52-6.66)	<0.05
Anxiety	0.548 (0.351)	1.73 (0.87-3.44)	0.119
Depression	-0.273 (0.502)	0.76 (0.28-2.04)	0.587

^*a*^Reference group: Stable Non-frail

^*b*^Modified Barthel Index (per 10-point decrease)

Across all trajectories, functional impairment (lower MBI) was the strongest and most consistent predictor of adverse trajectory membership (Table 2). For the “Rapidly Worsening Frailty” trajectory, the key predictors were lower MBI (OR = 9.34, 95% CI: 7.37–11.82, $p<0.05$), advanced age (OR = 1.07, $p<0.05$), and non-urban residence (OR = 0.34, $p<0.05$), while psychological factors were not significant in this group.

The “Slowly Progressive Frailty” trajectory was distinguished by the broadest set of predictors: in addition to functional impairment (MBI: OR = 8.23, $p<0.05$), psychological factors including anxiety (OR = 2.33, $p<0.05$) and depression (OR = 2.50, $p<0.05$), poor nutritional status (OR = 3.61, $p<0.05$), and behavioral factors were all significant.

The “Improving Frailty” trajectory shared several baseline risk factors with the adverse trajectories, including lower MBI (OR = 4.85, $p<0.05$), poor nutrition (OR = 4.24, $p<0.05$), and smoking/drinking history. However, psychological factors were not significant predictors for this group, distinguishing it from the Slowly Progressive trajectory.

These findings suggest that functional assessment should be prioritized for identifying high-risk patients, while psychological screening is particularly relevant for patients on a slowly progressive decline.

### Predictive model performance and clinical applicability

The multinomial logistic regression model achieved strong predictive performance for trajectory membership using baseline clinical features (Table 4 in Appendix A). The overall accuracy was 0.847 (95% CI: 0.814-0.880), with individual trajectory AUCs ranging from 0.823 to 0.941.

We also developed a clinical decision support framework that enables real-time patient classification and trajectory adjustment. The framework supports admission-based classification of patients into their most likely trajectory within 48 hours, enabling early care plan adjustment. At each subsequent follow-up (T2–T6), patient status is re-evaluated against trajectory-specific benchmarks to detect deviations and inform care plan modifications. Prediction confidence levels (low/medium/high) are included to guide the intensity of clinical follow-up.

## Discussion

In this prospective cohort study, we demonstrated that frailty progression following AMI is not a uniform process. Instead, by applying the SEC algorithm, we identified four frailty trajectories: “Rapidly Worsening Frailty”, “Slowly Progressive Frailty”, “Improving Frailty”, and “Stable Non-Frail”. These findings support the view that frailty after AMI follows heterogeneous clinical courses and that trajectory-based stratification is feasible.

Our central finding on the heterogeneity of frailty progression is consistent with recent literature emphasizing the importance of identifying distinct trajectories to guide clinical decision-making [13, 15, 30]. However, this study extends prior work by applying the SEC algorithm to multidimensional, longitudinal data. The SEC algorithm afforded a more interpretable patient stratification than traditional methods, enabling a finer characterization of the clinical features distinguishing each trajectory [22, 23, 31]. The identification of a small but critical ’Improving Frailty’ group, in particular, lends strong support to the concept that frailty is not invariably progressive, even within this high-risk AMI population, and supports the potential value of early, targeted interventions.

The results of this study have important implications for clinical nursing practice. A key implication is the potential to move toward a more stratified, evidence-based nursing approach, in contrast to the current “one-size-fits-all” care model.

- Early Identification of High-Risk Patients: Our finding that lower baseline functional status (MBI) and advanced age are the strongest predictors for the “Rapidly Worsening Frailty” trajectory suggests that simple, bedside assessments conducted at admission could support early identification of high-risk individuals. This may facilitate timely referral for multidisciplinary interventions, including nutritional support, physical rehabilitation, and psychological care [11].
- Optimizing Nursing Resource Allocation: By distinguishing between trajectories, healthcare organizations can allocate nursing resources more effectively. For instance, intensive rehabilitation resources can be prioritized for patients in the “Improving Frailty” group, who have high recovery potential. Meanwhile, long-term monitoring and preventive strategies can be designed for the “Slowly Progressive” group, and health promotion-focused care for the “Stable Non-Frail” cohort [10].
- Contributions to Clinical Decision Support: The identified trajectories and predictors can be developed and integrated into Electronic Health Record systems as a clinical decision support tool. Such a tool could automatically assess a newly admitted older AMI patient’s likely frailty trajectory, providing risk stratification information to nurses and physicians and facilitating the creation of personalized care plans [24, 25].

Beyond identifying trajectories, this study developed a predictive model for trajectory membership using admission-day clinical data. The multinomial logistic regression model demonstrated good predictive performance, with an accuracy of 84.7%, using readily available admission data. As key predictors such as MBI scores can be assessed within 48 hours of admission, these findings suggest potential for early clinical risk stratification. This could support nurses in identifying patients at risk of “Rapidly Worsening Frailty” (AUC: 0.941) and those with recovery potential in the “Improving Frailty” group (AUC: 0.823), thereby informing resource allocation. Prospective validation is needed before clinical deployment.

Beyond the initial admission prediction, our approach enables a dynamic care adjustment framework throughout the recovery process. This framework uses uncertainty quantification to provide confidence levels for each prediction, which in turn guides the intensity of clinical follow-up. For example, high confidence predictions (>80%) would lead to standard trajectory-specific care protocols, medium confidence predictions (60-80%) would prompt enhanced monitoring with monthly reassessment, and low confidence predictions (<60%) would trigger an intensive multidisciplinary evaluation and more frequent re-evaluation.

The primary strength of this study lies in its prospective, longitudinal design with six measurement time points, which allowed us to capture the dynamic nature of frailty over a full year of follow-up. The use of the SEC algorithm, a non-parametric method requiring no distributional assumptions, offers improved interpretability for complex clinical data compared to traditional clustering methods. The collection of physical, psychological, and functional measures at each time point enabled a detailed characterization of each trajectory.

However, several limitations must be acknowledged. First, this study was conducted at a single tertiary hospital using convenience sampling, which may introduce selection bias and limit the generalizability of the findings to broader or more diverse AMI populations. Second, the “Improving Frailty” subgroup comprised only 26 patients (4.5%), which may yield statistically unstable estimates with wide confidence intervals; findings for this trajectory should therefore be interpreted with caution. Third, the predictive model was evaluated exclusively through 5-fold cross-validation within the same cohort; in the absence of an independent external validation dataset, the risk of overfitting cannot be fully excluded. Fourth, the reasons for loss to follow-up were not systematically recorded; if attrition was associated with clinical outcomes, differential loss to follow-up could introduce informative censoring bias. Fifth, the Fried Frailty Phenotype captures physical frailty only, and cognitive, social, and psychological dimensions of frailty were not incorporated into the trajectory model. Sixth, the study was conducted in a single Chinese Han population, which may limit cross-cultural and cross-ethnic generalizability. Future studies should employ multicenter, consecutively enrolled designs across diverse healthcare settings and include independent validation cohorts to address these limitations.

These findings suggest several priorities for future research. First, multicenter validation studies across diverse healthcare settings and ethnic backgrounds are needed to confirm the stability and generalizability of the identified frailty trajectories. Second, randomized controlled trials should be designed and conducted to evaluate the clinical effectiveness and cost-efficiency of ’trajectory-specific’ nursing interventions. Finally, future work could explore the integration of real-time digital health monitoring technologies, such as wearable devices, into frailty management to dynamically track patient status and adjust interventions, thereby advancing the goal of personalized frailty management [15, 30].

## Conclusions

This study identified four frailty trajectories in older adults with AMI and characterized the baseline predictors associated with each. These findings provide evidence to support a shift toward more proactive and differentiated nursing strategies, enabling earlier identification of high-risk patients and more targeted resource allocation. Pending external validation, these results may contribute to improving long-term outcomes for this vulnerable population.

## Data Availability

De-identified data that support the findings of this study are available from the corresponding author upon reasonable request and with permission from the hospital’s ethics committee.

## Appendix A

**Table 3:** Baseline characteristics comparison between completed and dropout participants

Variable	Completed (n=583)	Dropout (n=32)	*P*-value
Demographic Characteristics			
Age, years	75.82 ± 6.08	75.62 ± 6.61	0.869
Sex			0.746
Female	283 (48.5%)	17 (53.1%)	
Male	300 (51.5%)	15 (46.9%)	
Residence			0.664
Rural	241 (41.3%)	15 (46.9%)	
Urban	342 (58.7%)	17 (53.1%)	
Family income			0.804
<3000 yuan	192 (32.9%)	9 (28.1%)	
3000-5000 yuan	190 (32.6%)	12 (37.5%)	
>5000 yuan	201 (34.5%)	11 (34.4%)	
Marital status			0.367
Married	383 (65.7%)	18 (56.2%)	
Unmarried	200 (34.3%)	14 (43.8%)	
Clinical Characteristics			
Charlson Comorbidity Index	1.59 ± 1.24	1.47 ± 1.34	0.626
Left ventricular ejection fraction, %	50.63 ± 6.42	51.03 ± 6.20	0.725
Cardiac function classification	1.87 ± 0.67	1.91 ± 0.53	0.697
Frailty phenotype count	2.15 ± 1.28	2.25 ± 1.19	0.662
Nutritional status			0.735
Normal	373 (64.0%)	19 (59.4%)	
Abnormal	210 (36.0%)	13 (40.6%)	
Anxiety			0.724
Normal	427 (73.2%)	22 (68.8%)	
Abnormal	156 (26.8%)	10 (31.2%)	
Depression			0.097
Normal	478 (82.0%)	30 (93.8%)	
Abnormal	105 (18.0%)	2 (6.2%)	
Other Characteristics			
Sleep score	7.60 ± 2.10	7.72 ± 2.32	0.773
Activities of daily living score	74.24 ± 24.19	77.66 ± 23.32	0.427
Drinking history			0.908
No	452 (77.5%)	24 (75.0%)	
Yes	131 (22.5%)	8 (25.0%)	
Smoking history			0.400
No	481 (82.5%)	24 (75.0%)	
Yes	102 (17.5%)	8 (25.0%)	
Fall history			0.371
No	422 (72.4%)	26 (81.2%)	
Yes	161 (27.6%)	6 (18.8%)

**Table 4:** Predictive model performance for frailty trajectory classification

Trajectory	Precision	Recall	F1-Score	AUC
Rapidly Worsening Frailty	0.834	0.821	0.827	0.941
Slowly Progressive Frailty	0.856	0.849	0.852	0.889
Improving Frailty	0.788	0.769	0.778	0.823
Stable Non-Frail	0.872	0.888	0.880	0.924
Overall Accuracy	0.847 (95% CI: 0.814-0.880)

**Table 5:** Complete dataset variable classification

Variable name	Type
Static Variables (Time-Invariant, T1, n=8)	
Age	Static
Sex	Static
Residence	Static
Income	Static
Marital Status	Static
Drinking History	Static
Smoking History	Static
Fall History	Static
Longitudinal Variables (Time-Varying, T1-T6, n=12)	
Long-term Medication	Longitudinal
Charlson Comorbidity Index	Longitudinal
Nutrition Status	Longitudinal
Sleep Score	Longitudinal
Modified Barthel Index	Longitudinal
Anxiety	Longitudinal
Depression	Longitudinal
Comorbidity Count	Longitudinal
Stent Count	Longitudinal
Left Ventricular Ejection Fraction	Longitudinal
Killip Classification	Longitudinal
Fried Frailty Phenotype Count	Longitudinal

## Abbreviations

AMI: Acute myocardial infarction
SEC: Structural entropy clustering
CCI: Charlson comorbidity index
LVEF: Left ventricular ejection fraction
MBI: Modified Barthel Index
DTW: Dynamic time warping
OR: Odds ratio
CI: Confidence interval
STROBE: Strengthening the Reporting of Observational Studies in Epidemiology
ANOVA: Analysis of variance
HSD: Honest significant difference
AUC: Area under the receiver operating characteristic curve
SD: Standard deviation
MICE: Multiple imputation with chained equations

## Code availability

The code used during the current study is available from the corresponding author on reasonable request.
